# *Tenebrio molitor* and *Zophobas morio* Full-Fat Meals in Broiler Chicken Diets: Effects on Nutrients Digestibility, Digestive Enzyme Activities, and Cecal Microbiome

**DOI:** 10.3390/ani9121128

**Published:** 2019-12-12

**Authors:** Abdelbasset Benzertiha, Bartosz Kierończyk, Mateusz Rawski, Agata Józefiak, Krzysztof Kozłowski, Jan Jankowski, Damian Józefiak

**Affiliations:** 1 Department of Animal Nutrition, Poznań University of Life Sciences, Wołyńska 33, 60-637 Poznań, Poland or bartosz.kieronczyk@up.poznan.pl (B.K.); 2 HiProMine S.A., Poznańska 8, 62-023 Robakowo, Poland; 3 Division of Inland Fisheries and Aquaculture, Institute of Zoology, Poznań University of Life Sciences, Wojska Polskiego 71c, 60-625 Poznań, Poland; mateusz.rawski@up.poznan.pl; 4 Department of Preclinical Sciences and Infectious Diseases, Poznań University of Life Sciences, Wołyńska 35, 60-637 Poznań, Poland; agata.jozefiak@up.poznan.pl; 5 Department of Poultry Science, Faculty of Animal Bioengineering, University of Warmia and Mazury in Olsztyn, Oczapowskiego 5, 10-719 Olsztyn, Poland; kristof@uwm.edu.pl (K.K.); janj@uwm.edu.pl (J.J.)

**Keywords:** insect meals, poultry, feed formulation, gut microbiota, pancreatic enzymes

## Abstract

**Simple Summary:**

In animal nutrition, the links among health status, alimentary tract factors and gastrointestinal tract (GIT) integrity are increasing in importance. It has been documented that insects are useful as novel ingredients in poultry diets because they contain bioactive compounds such as chitin, melanin, and antimicrobial peptides, in addition to protein and dietary fat, and these bioactive compounds have been reported to modulate the GIT microbiome. From this perspective, insects could be promising functional feed additives to stimulate GIT health through inhibition of potentially pathogenic bacteria. Therefore, we evaluated the effect of the addition of a small amount of insects to the broiler chicken diet on the GIT microbiota composition and activity. Six experimental groups were used in the current study, i.e., two different levels (0.2% and 0.3%) of yellow mealworm and super mealworm full-fat meals, a positive control with salinomycin addition, and a negative control without any additives. Insect full-fat meals were capable of improving the health status of the birds by a positive reduction in potentially pathogenic bacteria in the cecal digesta and stimulation of the GIT microbiome to produce enzymes, especially glycolytic enzymes.

**Abstract:**

This study was conducted to investigate the effect of insect full-fat meals added in relatively small amounts to a complete diet on the coefficients of apparent ileal digestibility, short-chain fatty acid (SCFA) concentrations, bacterial enzymes, and the microbiota community in the cecal digesta of broiler chickens. In total, 600 one-day-old female Ross 308 broiler chicks were randomly assigned to six dietary treatments with 10 replicate pens/treatment and 10 birds/pen. The groups consisted of a negative control (NC) with no additives; a positive control (PC; salinomycin 60 ppm), and supplementation with 0.2% or 0.3% *Tenebrio molitor* or *Zophobas morio* full-fat meals. *Z. morio* (0.2%) addition increased the activities of α- and β-glucosidase and α-galactosidase. Dietary insects significantly decreased the cecal counts of the *Bacteroides–Prevotella* cluster in comparison to those in the NC and PC. Whereas, *Clostridium perfringens* counts were increased in the broiler chickens subjected to the 0.3% *Z. morio* treatment. In conclusion, small amounts of full-fat insect meals added to broiler diets were capable of reducing the abundance of potentially pathogenic bacteria, such as the *Bacteroides–Prevotella* cluster and *Clostridium perfringens*. In addition, this supplementation was able to stimulate the GIT microbiome to produce enzymes, especially glycolytic enzymes.

## 1. Introduction

Gastrointestinal tract (GIT) microbiota modulation is one of the key factors in achieving high growth performance and healthy broiler chickens. Improvement of intestinal integrity can exert a crucial role in regulating physiological homeostasis as well as supporting host resistance to infectious agents [1]. There appears to be an interaction among diet, GIT microbiota, and the immune system with a direct impact on growth performance [2]. Diet composition and its physical structure were found to be one of the factors influencing intestinal bacterial composition and activity in broilers [3,4,5]. In addition, the GIT microbiota plays an important role in improving and maintaining the innate immune system of broiler chickens, which has a positive effect on growth performance [5,6]. The cecum contains the highest microbial cell densities and plays an important role through the fermentation of nutrients to produce short-chain fatty acids (SCFAs). These fatty acids are transferred to the blood and thus can contribute energy to the body [7,8,9]. Recently, insects have been proposed as an alternative source of protein and fat for broilers, turkeys and laying hens [10,11,12,13,14]. In addition, insects were found to be one of the nutrient factors that can modulate the GIT microbiota populations during full or partial replacement of protein sources [15,16,17,18,19] and as feed additives in the diet of broiler chickens [20]. This microbiota modulation can be achieved through bioactive compounds that are present in insects, i.e., chitin, and antimicrobial peptides (AMPs) [16,20]. Yellow mealworm (*Tenebrio molitor*) and super mealworm larvae (*Zophobas morio*) presented an interesting nutrient profile, with 451 to 603 g/kg of dry matter (DM) of protein and 250 to 431 g/kg of DM of fat content [11]. Insect exoskeletons contain a large amount of chitin. Moreover, chitin was reported to have a beneficial effect on the innate immune system, and its antibacterial activity has been intensively studied [21,22,23]. However, De Marco et al. [24] and Schiavone et al. [25] reported that insects’ chitin might have negative effects on the coefficients of apparent ileal digestibility of crude protein in broiler chicken. The current study is a continuation of a previous trial described in Benzertiha et al. [26], in which insect full-fat meals that were added in small amounts to a complete diet of broiler chickens had positive effects on growth. In brief, the body weight gain and feed intake were increased in dietary groups supplemented with insect full-fat meals while comparing to positive control (with salinomycin addition, 60 ppm) and negative control (without any additions). The feed conversion ratio was not affected by any of the dietary treatments. In the previous study, insect full-fat meals positively affected the level of plasma immunoglobulins, i.e., IgY and IgM. However, despite the growing research interests in insect application in poultry nutrition, information about supplementation is still sparse for broiler chickens. Thus, the present study aimed to evaluate the effect of *T. molitor* and *Z. morio* full-fat meals added in small amounts (0.2% and 0.3%) to a complete diet on the coefficients of apparent ileal digestibility, pancreatic enzyme activity, short-chain fatty acid concentrations, bacterial enzymes, and microbiota community in the cecal digesta of broiler chickens.

## 2. Materials and Methods

According to Polish law and the EU directive (no. 2010/63/EU), the experiment carried out does not require the approval of the Local Ethics Committee for Experiments on Animals in Poznań. However, all animals were treated humanely according to the guidelines, and all efforts were made to minimize animal suffering.

### 2.1. Birds and Housing

The current trial was conducted at the experimental station unit (Piast, Olszowa Experimental Unit, no. 0161, Poland). A total of 600 one-day-old female Ross 308 broiler chicks were reared till 35 days of age. The birds were randomly distributed to six dietary treatments with 10 replicate pens per treatment, each consisting of 10 birds. Each replicate was placed in a floor pen (1.00 × 1.00 m). Further, the birds were reared in a chicken house according to AVIAGEN guidelines. The temperature and lighting regime met commercial recommendations. Vaccination against Gumboro disease was done for all birds at day 21 (AviPro PRECISE, Lohmann Animal GmbH, Cuxhaven, Germany).

### 2.2. Diets and Feeding Program

The ingredients and calculated nutritive value of the basal diet are presented in Table 1. The birds were offered mash form diet ad libitum for the whole period of the trial. All the raw materials were ground by a disc mill (Skiold A/S, Saby, Denmark) at a 2.5-mm disc distance and mixed without the application of any heat treatment. The diets were produced in accordance with ISO 9001:2008 procedures in a feed mill (Piast Pasze, Lewkowiec, Poland). The diets were prepared on a laboratory-scale line equipped with a horizontal double band mixer (Zuptor, Gostyń, Poland) with roller mills (Skiold, Saby, Denmark). The birds were offered a starter diet from 1 to 14 days of age and grower from 15 to 35 days of age. The nutritive values of the basal diets (starter and grower) were calculated to meet or exceed nutrients requirements of broilers as required by Nutrient Requirements of Poultry (NRC) [27]. Exogenous enzymes were not added to the diets. From 30 to 35 days of age, 0.2% of wheat in the diets was replaced by titanium dioxide (TiO_2_), which was used as an internal marker for calculation of nutrients digestibility. The insect full-fat meals were applied “on top” of the complete diet, and the experimental groups were as follows: PC (positive control)—NC + salinomycin addition (60 ppm); NC (negative control)—no additives; TM02—NC + 0.2% *T. molitor* full-fat meal; ZM02—NC + 0.2% *Z. morio* full-fat meal; TM03—NC + 0.3% *T. molitor* full-fat meal; and ZM03—NC + 0.3% *Z. morio* full-fat meal.

### 2.3. Preparation of Insect Full-Fat Meals

*T. molitor* and *Z. morio* used in the current trial were purchased from a commercial source (HiProMine S.A., Robakowo, Poland), air-dried in an oven (SLN 240, POL–EKO Aparatura, Wodzisław Śląski, Poland) for 24 h at 50 °C, and to obtain full-fat meals the dried form of insects were ground (Zelmer Motor Blocked Power 1900 W, Rzeszów, Poland). The analyzed compositions of both insect full-fat meals used in the current study are presented in Table 2.

### 2.4. Data and Sample Collection

At the end of the experiment (35 days), one bird from each replication (10 birds per group) were randomly selected and killed by cervical dislocation. The crop, jejunum, and ceca were gently emptied, and digesta was collected for measurements of pH, bacterial enzymes, and organic acid concentrations. The ileum was identified between the Meckel’s diverticulum and the ileocecal-colonic junction, and its contents were collected to perform the analysis of coefficients of apparent ileal digestibility of crude protein (CP), ether extract (EE), and apparent metabolizable energy (AME_N_). Furthermore, the duodenal content was collected for pancreatic enzyme activity analysis. Immediately after collection, all samples were stored at −80 °C for further analysis.

### 2.5. Chemical Analysis and Digestibility Determination

The nutrient composition of the diets, digesta, as well as insect meals, were analyzed, as described by the Association of Official Analytical Chemists (AOAC) [28] using the methods 934.01, 976.05, 920.39, 942.05, and 985.29 for dry matter (DM), CP, EE, crude ash, and crude fiber (CF), respectively. Calcium and phosphorus in insect meals were determined according to the procedures described by Ptak et al. [29]. Further, the chitin content of the insect meals was analyzed, as presented by Soon et al. [30]. TiO_2_ analysis was performed according to Myers et al. [31], and the concentration was estimated, as described by Short et al. [32]. Gross energy (GE) was analyzed using an adiabatic bomb calorimeter (KL 12 Mn, Precyzja-Bit PPHU Sp. z o.o., Bydgoszcz, Poland) standardized with benzoic acid.

The coefficients of apparent ileal digestibility of CP and EE were calculated relative to the ratio of titanium dioxide (TiO_2_) to the nutrient content in the feed or digesta. The relative N retention coefficient was determined, as shown by Kaczmarek et al. [33]. The following equation was used (CP digestibility calculation is used as an example):

$${\mathrm{Digestibility}\;}_{\;\mathrm{crude}\;\mathrm{protein}}=\left(1-\left[\left(\frac{{\mathrm{TiO}}_{2\frac{g}{\;\mathrm{kg}\;\mathrm{diet}}}}{{\mathrm{TiO}}_{2\;\frac{g}{\mathrm{kg}}\;\mathrm{digesta}}}\right)\times \left(\frac{{\mathrm{Crude}\;\mathrm{protein}\;}_{\frac{g}{\mathrm{kg}}\;\mathrm{digesta}}}{{\mathrm{Crude}\;\mathrm{protein}}_{\frac{g}{\mathrm{kg}}\;\mathrm{diet}}}\right)\right]\right)$$

### 2.6. Analysis of pH and Pancreatic Enzyme Activity

The pH value of the crop, jejunal, and cecal content was measured using a combined glass and reference electrode (pH 100 L; VWR International, Leuven, Belgium). The activity of pancreatic enzymes was analyzed, as presented in detail by Pruszyńska-Oszmałek et al. [34].

### 2.7. Analysis of Fermentation Products and Bacterial Enzyme Activities in the Ceca

Short-chain fatty acids in the cecal digesta samples were analyzed, as described in detail by Fotschki et al. [35]. The activity of extracellular bacterial enzymes in the cecal digesta was measured, as presented in detail by Juśkiewicz et al. [36]

### 2.8. Microbial Community Analysis by Fluorescent In Situ Hybridization (FISH)

The microbial community of the cecal content of chickens was analyzed, as described in detail by Józefiak et al. [37] and Rawski et al. [38]. The oligonucleotide probes used for this study are shown in Table 3.

### 2.9. Calculations and Statistical Analysis

The designs of the experiments were completely randomized, and data were tested using the General Linear Models procedure of SAS software (SAS Institute Inc., Cary, NC, USA). In the experiments, means were separated using Duncan’s tests following one-way ANOVA based on the following equation:

$$y_{ij}=\;\mu +\;a_{i}+\delta_{ij}$$

where *y_ij_* is the observed dependent variable; *μ* is the overall mean; α*_i_* is the effect of treatment; and *δ_ij_* is the random error. In cases in which the overall effect was significant, *p* ≤ 0.05.

## 3. Results

### 3.1. Coefficients of Apparent Ileal Digestibility and Pancreatic Enzyme Activity

The effect of insect full-fat meals on the coefficients of apparent ileal digestibility of CP, EE, and AME_N_ is shown in Table 4. The apparent ileal digestibility of CP, EE, and AME_N_ was not affected (*p* > 0.05) by any of the dietary treatments. Moreover, the activity of pancreatic enzyme activity (lipase, amylase, and trypsin) did not differ significantly (*p* > 0.05) (Table 4).

### 3.2. Gastrointestinal Tract Content pH

The pH values of the crop, jejunal, and cecal content were not affected (*p* > 0.05) by any of the dietary treatments (Table 5).

### 3.3. Microbial Fermentation Patterns and Enzyme Activities

The activity of extracellular enzymes in the cecal digesta is shown in Table 6. In general, dietary treatments affected specific enzymes. The highest α-glucosidase activity was observed in TM03 and ZM02; however, the lowest activity was noted in NC (*p* = 0.004). β-glucosidase was also affected (*p* = 0.001); PC decreased its activity compared to TM02 and ZM02, while the highest activity was observed in ZM02 compared to PC, NC, TM03, and ZM03. Compared to other treatments, the addition of ZM02 resulted in the highest α-galactosidase and β-glucuronidase activities in the cecal content (*p* < 0.001). No effect (*p* > 0.05) on β-galactosidase was observed. Furthermore, α-arabinopyranosidase was also affected (*p* < 0.001), and its activity was highest in the NC group. β-xylosidase showed the highest activity in the ZM02 group.

The short-chain fatty acid concentrations in the cecal digesta were not affected (*p* > 0.05) by any of the dietary supplements (Table 7).

### 3.4. Microbial Community Analysis

Dietary treatment did not show any effect (*p* > 0.05) on the total number of bacteria (Table 8). Compared to NC and PC, dietary insect full-fat meals significantly (*p* = 0.001) decreased the cecal population of the *Bacteroides-Prevotella* cluster, among which ZM02 showed the lowest value. Moreover, in the ZM03 treatment, *Clostridium perfringens* showed the highest values (*p* = 0.033) compared to those in the other treatments, and the lowest value was observed in TM02. In addition, dietary treatments did not show any significant effect (*p* > 0.05) on the *Clostridium leptum* subgroup, *Clostridium coccoides–Eubacterium rectale* cluster, *Lactobacillus* spp./*Enterococcus* spp., and Enterobacteriaceae counts.

## 4. Discussion

Several studies have reported the interaction between the immune system and the GIT microbiota [1,8,45,46]. Therefore, the present study represents an evaluation of cecal microbial community modulation and SCFA production, as well as of the activity of extracellular bacterial enzymes in the cecal digesta after 0.2% and 0.3% administration of insect full-fat meals in the diet of broiler chickens.

In the current findings, insect full-fat meals did not show any negative effects on the ileal digestibility coefficients of CP, EE, or AME_N_. Schiavone et al. [25] reported that the ileal digestibility coefficient of CP was lowered in a group fed 25% *T. molitor* larvae meal compared to that in a group fed soybean meal. The same effect was shown by Bovera et al. [47]. The authors related this effect to the high chitin content of the insect exoskeleton, which might have a negative impact on the ileal digestibility coefficients. In the present study, the chitin content of *T. molitor* and *Z. morio* was determined at 8.91% of DM and 4.59% of DM, respectively. This finding of the chitin content of both insect species is in agreement with the results of Bovera et al. [47] and Finke [48]. It is possible that due to the low inclusion level of both insect species in the diet, no negative effect on the ileal digestibility coefficient of nutrients was observed. Furthermore, analysis of the selected pancreatic enzymes confirmed the abovementioned results, and no effect on their activity was demonstrated. According to Józefiak et al. [20,49,50], the pH values of the digesta can be affected by dietary factors in different parts of the gastrointestinal tract (crop, jejunum, and cecum). Józefiak et al. [20] recorded that the pH value of the crop content was significantly reduced after supplementation of a low amount of insect full-fat meals (0.1% and 0.2%) in broiler chickens’ diet. However, in the present experiment, opposite results were observed. This discrepancy could be explained by the small (0.2% and 0.3%) insect full-fat meal addition amount used in this study.

Insect meal was reported to affect the SCFA content in the cecal digesta of laying hens, as well as in that of broiler chickens. Borrelli et al. [17] showed that a full replacement of soybean meal by *Hermetia illucens* larvae meal in the hen diet led to an increased production of butyric acid in the ceca, and the authors related the changes to modification of the microbiota. Furthermore, Loponte et al. [18] reported that broilers fed *T. molitor* meal as a full replacement of soybean meal showed increased SCFA levels in the cecal digesta, in which butyrate showed an increase of 185% compared to the level in the soybean meal group. The authors related this effect to the chitin content in the insect full-fat meals. In our study, the addition of a small amount of insect full-fat meal did not show any effect on the concentration of SCFAs in the ceca.

The replacement of soybean meal with insect meal as a source of protein has been reported to modulate the GIT microbiota population in broiler chickens and laying hens [15,16,17,18,19,20]. Our findings using insects as feed additives did not show any effects on the total number of bacteria. However, insect meal addition decreased the level of the *Bacteroides–Prevotella* cluster compared to that in the NC and PC. These results are in agreement with a study conducted by Józefiak et al. [20], in which the *Bacteroides–Prevotella* cluster level was lowered with the inclusion of 0.2% *T. molitor* full-fat meal. Furthermore, Biasato et al. [15] found that *Bacteroides* abundance was lowered in the ceca of broilers fed diets in which soybean meal was replaced by *T. molitor* meal. The reduction in *Bacteroides* abundance in the cecal content of the broiler chickens fed insect full-fat meal in the current study may be considered a potential positive effect. *Bacteroides* is one of the most commonly isolated pathogenic genera from clinical specimens [51]. Furthermore, *C. perfringens* is one of the most pathogenic bacteria in poultry production, causing necrotic enteritis [50,52,53,54]. *T. molitor* (0.2% and 0.3%) and *Z. morio* at 0.2% inclusion decreased the level of *C. perfringens*. Despite the overall effect, *T. molitor* showed a positive effect against *C. perfringens*.

The activity of the glycolytic enzymes α- and β-glucosidase and α-galactosidase was affected by the dietary treatments. ZM02 showed an increase in their activity. High activity of α-galactosidase and α-glucosidase may enhance the fermentation of lactose, raffinose, and resistant starch, which may lead to the production of SCFAs and lactic acids, which are sources of energy for GIT tissues [55]. On the other hand, the β-glucuronidase and β-glucosidase activity levels are often used to determine the pathogenic microbiota activity causing undesirable metabolic changes [56]. β-glucuronidase activity was low in treatments with salinomycin and *T. molitor* addition at both levels (0.2% and 0.3%), which may be considered positive findings because the high activity of this enzyme is potentially harmful to the host due to its involvement in the regeneration of toxic and carcinogenic metabolites in the hindgut [57]. Djouzi and Andiueux [58] reported that neither the decrease in the pH nor the changes in bacterial composition were sufficient to explain the glycolytic activity variations. We suggest that the small addition of insect full-fat meal to the diet of broiler chickens was enough to stimulate the GIT microbiome to produce enzymes, especially glycolytic enzymes.

## 5. Conclusions

Insect full-fat meals derived from *T. molitor* and *Z. morio* added in a small amount to the complete diet of broiler chickens did not have any negative effects on the nutrient ileal digestibility coefficients or the activity of pancreatic enzymes. Furthermore, dietary insect full-fat meals were capable of improving the health status of the birds by reducing pathogenic bacterial concentrations, such as those of the *Bacteroides–Prevotella* cluster and *C. perfringens*. In addition, this small amount of supplementation stimulated the GIT microbiota to produce enzymes, especially glycolytic enzymes.

## Figures and Tables

**Table 1:** Composition of the basal experimental diets.

Ingredients (%)	1–14 Days	15–35 Days
Wheat	48.74	51.34
Soybean meal	20.78	16.95
Rye	10.00	10.00
Rapeseed meal	10.00	10.00
Soybean oil	4.99	7.11
Fish meal	2.00	2.00
Monocalcium phosphate	1.31	0.67
Limestone	0.8	0.68
Vitamin–mineral premix ^a^	0.3	0.3
Methionine 88% liquid	0.31	0.25
L–Lysine HCl	0.29	0.24
Sodium carbonate (Na_2_CO_3_)	0.22	0.17
L–Threonine	0.15	0.16
Salt (NaCl)	0.11	0.13
Titanium dioxide (TiO_2_) ^b^	-	0.2
Calculated nutritive value (%)		
Crude protein	21.56	20.06
Ether extract	6.54	8.63
Crude fiber	3.31	3.22
Calcium (Ca)	0.85	0.70
Total phosphorus (P)	0.79	0.63
Lysine	1.25	1.12
Methionine	0.61	0.53
Methionine + cysteine	0.99	0.90
Threonine	0.91	0.86
AME_N_ (MJ·kg^−1^)	12.56	13.31

^a^ Provided per 1 kg of diet: vitamin A, 11,166 IU; vitamin D_3_, 2500 IU; vitamin E, 80 mg; menadione, 2.50 mg; vitamin B_1_, 2.17 mg; vitamin B_2_, 7.0 mg; vitamin B_6_, 4.0 mg; vitamin B_7_, 0.18 mg; vitamin B_9_, 1.17 mg; vitamin B_12_, 0.02 mg; choline, 379 mg; D–pantothenic acid, 12.50 mg; niacin, 41.67 mg; ethoxyquin, 0.09 mg; Mn (MnO_2_), 73 mg; Zn (ZnO), 55 mg; Fe (FeSO_4_), 45 mg; Cu (CuSO_4_), 20 mg; I (CaI_2_O_6_), 0.62 mg; and Se (Na_2_SeO_3_), 0.3 mg. ^b^ Replaced the corresponding amount of wheat in each diet from 30 to 35 days of broiler growth.

**Table 2:** Nutrient composition of *Tenebrio molitor* and *Zophobas morio* full-fat meals used in the experiment (g kg^−1^ of DM).

Items	*Tenebrio molitor*	*Zophobas morio*
Dry matter (%)	95.58	96.32
Crude protein	470	493
Ether extract	296	336
Crude ash	25.6	25.2
Crude fiber	56.0	51.0
Chitin	89.1	45.9
Calcium	0.5	0.5
Phosphorus	7.2	6.2

**Table 3:** Oligonucleotide probes used for cecal microbiota analysis using fluorescent in situ hybridization (FISH).

Target	Probe	Sequence (5′ to 3′)	References
*Bacteroides–Prevotella* cluster	Bacto303	CCAATGTGGGGGACCTT	[39]
*Clostridium perfringens*	Cperf191	GTAGTAAGTTGGTTTCCTCG	[40]
Enterobacteriaceae	Enter1432	CTTTTGCAACCCACT	[41]
*Lactobacillus* spp./*Enterococcus* spp.	Lab158	GGTATTAGCAYCTGTTTCCA	[42]
*Clostridium coccoides–Eubacterium rectale* cluster	Erec482	GCTTCTTAGTCARGTACCG	[43]
*Clostridium leptum* subgroup	Clept1240	GTTTTRTCAACGGCAGTC	[44]

**Table 4:** Coefficients of apparent ileal digestibility of crude protein, ether extract, and apparent metabolizable energy corrected to zero nitrogen balance, as well as activities of selected pancreatic enzymes in the duodenal digesta of broiler chickens, expressed as % of control.

Items	Treatments							
	PC	NC	TM02	ZM02	TM03	ZM03	SEM	*p*-Value
Coefficients of apparent ileal digestibility								
CP	0.73	0.76	0.75	0.75	0.73	0.77	0.03	0.304
EE	0.92	0.94	0.94	0.94	0.93	0.94	0.01	0.092
AME_N_ (MJ)	10.64	12.05	11.95	12.04	11.37	11.87	0.91	0.140
Activity of pancreatic enzymes								
Lipase	100	88.33	93.23	91.12	87.13	88.33	32.431	0.958
Amylase	100	91.73	103.44	118.81	151.10	240.76	161.67	0.322
Trypsin	100	88.9	100.67	95.04	87.48	104.87	49.224	0.962

PC—positive control (salinomycin, 60 ppm); NC—negative control (no additives); TM02—(0.2% *T. molitor* full-fat meal); ZM02—(0.2% *Z. morio* full-fat meal); TM03—(0.3% *T. molitor* full-fat meal); ZM03—(0.3% *Z. morio* full-fat meal); SEM—standard error of the mean; CP—crude protein; EE—ether extract.

**Table 5:** The effect of dietary supplementation with insect meals on the pH value of the gastrointestinal tract (GIT) content.

Items	Treatments							
	PC	NC	TM02	ZM02	TM03	ZM03	SEM	*p*-Value
Crop	4.48	4.17	4.64	4.48	4.7	4.62	0.06	0.071
Jejunum	5.81	5.91	5.96	5.99	6.04	6.03	0.03	0.102
Cecum	5.81	6.11	6.17	6.07	6.08	6.05	0.07	0.802

PC—positive control (salinomycin, 60 ppm); NC—negative control (no additives); TM02—(0.2% *T. molitor* full-fat meal); ZM02—(0.2% *Z. morio* full-fat meal); TM03—(0.3% *T. molitor* full-fat meal); ZM03—(0.3% *Z. morio* full-fat meal); SEM—standard error of the mean.

**Table 6:** Activity of extracellular bacterial enzymes in the cecal digesta (µmol/h/g digesta).

Items	Treatments							
	PC	NC	TM02	ZM02	TM03	ZM03	SEM	*p*-Value
α^_^glucosidase	14.53 ^ab^	10.99 ^c^	14.29 ^ab^	15.97 ^a^	19.10 ^a^	12.58 ^bc^	3.14	0.004
β^_^glucosidase	1.53 ^c^	1.89 ^bc^	2.45 ^ab^	3.22 ^a^	1.87 ^bc^	1.62 ^bc^	0.91	0.001
α^_^galactosidase	10.57 ^b^	7.36 ^b^	8.00 ^b^	15.09 ^a^	9.78 ^b^	9.46 ^b^	3.66	<0.001
β^_^galactosidase	25.17	22.59	22.67	29.66	25.9	22.79	7.1	0.199
β^_^glucuronidase	3.01 ^c^	5.73 ^ab^	4.17 ^bc^	6.51 ^a^	4.23 ^bc^	5.14 ^ab^	2.13	0.009
α^_^arabinopyranosidase	0.84 ^c^	1.88 ^a^	1.07 ^bc^	1.66 ^ab^	1.59 ^ab^	0.67 ^c^	0.66	<0.001
β^_^xylosidase	1.35 ^b^	1.71 ^b^	2.23 ^ab^	2.69 ^a^	1.84 ^ab^	1.35 ^b^	0.9	0.017

PC—positive control (salinomycin, 60 ppm); NC—negative control (no additives); TM02—(0.2% *T. molitor* full-fat meal); ZM02—(0.2% *Z. morio* full-fat meal); TM03—(0.3% *T. molitor* full-fat meal); ZM03—(0.3% *Z. morio* full-fat meal); SEM—standard error of the mean; ^a–c^ means within a row with no common superscripts differ significantly (*p ≤* 0.05).

**Table 7:** Short-chain fatty acids concentration and profile in the cecal digesta of broiler chickens.

Items	Treatments							
	PC	NC	TM02	ZM02	TM03	ZM03	SEM	*p*-Value
SCFA concentration (µmol/g digesta)								
Acetic acid	67.79	58.73	62.82	61.97	63.09	57.13	15.17	0.695
Propionic acid	7.09	5.49	5.44	5.5	5.36	4.8	1.79	0.071
Butyric acid	13.07	15.05	15.09	14.21	13.63	13.19	5.93	0.949
Valeric acid	1.25	0.96	1.12	1.02	1.07	0.92	0.4	0.497
Iso-valeric acid	0.62	0.48	0.59	0.55	0.55	0.58	0.22	0.803
Iso-butyric acid	0.58	0.36	0.4	0.42	0.42	0.4	0.21	0.249
PSCFA	2.44	1.8	2.11	1.99	2.03	1.89	0.67	0.359
sum SCFA	90.4	80.43	85.45	83.65	84.1	77	20.07	0.765
SCFA profile (% of total SCFA)								
Acetic acid profile	75.17	73.05	73.78	74.27	75.31	73.6	4.02	0.774
Propionic acid profile	7.87	6.61	6.61	6.53	6.45	7.22	2.56	0.793
Butyric acid profile	14.35	17.91	17.1	16.81	15.84	16.22	5.11	0.720

PC—positive control (salinomycin, 60 ppm); NC—negative control (no additives); TM02—(0.2% *T. molitor* full-fat meal); ZM02—(0.2% *Z. morio* full-fat meal); TM03—(0.3% *T. molitor* full-fat meal); ZM03—(0.3% *Z. morio* full-fat meal); SEM—standard error of the mean; PSCFA—putrefactive short-chain fatty acid; SCFA–short-chain fatty acid.

**Table 8:** Selected microbiota counts in the cecal digesta (log CFU/g of digesta) determined by DAPI (4’,6-diamidino-2-phenylindole) staining and fluorescent *in situ* hybridization (FISH).

Items	Treatments							
	PC	NC	TM02	ZM02	TM03	ZM03	SEM	*p*-Value
Total number of bacteria	10.24	10.16	10.19	10.19	10.22	10.15	0.07	0.254
*Bacteroides–Prevotella* cluster	9.52 ^a^	9.53 ^a^	9.45 ^ab^	9.26 ^c^	9.37 ^bc^	9.34 ^bc^	0.1	0.001
*Clostridium leptum* subgroup	9.32	9.22	9.29	9.22	9.36	9.32	0.16	0.648
*Clostridium perfringens*	9.4 ^ab^	9.37 ^b^	9.35 ^b^	9.31 ^b^	9.36 ^b^	9.54 ^a^	0.11	0.033
*Clostridium coccoides*–*Eubacterium rectale* cluster	9.41	9.31	9.39	9.4	9.46	9.39	0.13	0.663
*Lactobacillus* spp./*Enterococcus* spp.	9.38	9.34	9.33	9.29	9.34	9.46	0.14	0.543
Enterobacteriaceae	9.22	9.32	9.16	9.24	9.43	9.4	0.17	0.140

PC—positive control (salinomycin, 60 ppm); NC—negative control (no additives); TM02—(0.2% *T. molitor* full-fat meal); ZM02—(0.2% *Z. morio* full-fat meal); TM03—(0.3% *T. molitor* full-fat meal); ZM03—(0.3% *Z. morio* full-fat meal); SEM—standard error of the mean; ^a–c^ means within a row with no common superscripts differ significantly (*p ≤* 0.05).
